# High-density Surface and Intramuscular EMG Data from the Tibialis Anterior During Dynamic Contractions

**DOI:** 10.1038/s41597-023-02114-1

**Published:** 2023-07-06

**Authors:** J. Cortney Bradford, Andrew Tweedell, Logan Leahy

**Affiliations:** 1 US Army DEVCOM Army Research Laboratory, Aberdeen Proving Ground, USA; 2 US Army Military Intelligence Corps., Fort Belvoir, USA

**Keywords:** Brain-machine interface, Neurophysiology

## Abstract

Valid approaches for interfacing with and deciphering neural commands related to movement are critical to understanding muscular coordination and developing viable prostheses and wearable robotics. While electromyography (EMG) has been an established approach for mapping neural input to mechanical output, there is a lack of adaptability to dynamic environments due to a lack of data from dynamic movements. This report presents data consisting of simultaneously recorded high density surface EMG, intramuscular EMG, and joint dynamics from the tibialis anterior during static and dynamic muscle contractions. The dataset comes from seven subjects performing three to five trials each of different types of muscle contractions, both static (isometric) and dynamic (isotonic and isokinetic). Each subject was seated in an isokinetic dynamometer such that ankle movement was isolated and instrumented with four fine wire electrodes and a 126-electrode surface EMG grid. This data set can be used to (i) validate methods for extracting neural signals from surface EMG, (ii) develop models for predicting torque output, or (iii) develop classifiers for movement intent.

## Background & Summary

Wearable human-machine interfaces designed to enhance human physical capabilities, such as robotic exoskeletons and prosthetics, have made rapid progress in recent years, but how the machine optimally anticipates the human’s intent to move remains a significant problem^1^. One established approach for the neural control of wearable robotics is using electrical signals derived from muscles via electromyography (EMG)^2,3^. EMG activity recorded at the skin surface is the summation of all neural signals instructing the muscle to generate force and thus includes information pertaining to an individual’s intent to move. This movement intent has the potential for utilization as a control mechanism that is responsive to dynamic environments. While there has been some initial success with EMG controlled prosthetics in patient populations^4^, the coarse nature of traditional surface EMG recordings has limited the development of fully functional prosthetics or wearable robotics^5^.

Muscles are composed of many motor units which are groups of muscle fibers innervated by a single motor neuron. The central nervous system controls muscle force generation via the recruitment of motor units and the action potential discharge rates of the recruited motor units^6^. There is a very reliable connection between the nerve and muscle, such that almost every action potential discharged by the motor neuron results in muscle fiber action potentials. Thus, the electrical activity generated by the muscle is representative of the motor commands sent to the muscle via the motor neuron pool.

Surface EMG only provides a coarse measure of the neural commands being sent to the muscle due to tissue filtering effects, artifacts, and amplitude cancellation, as the recorded signal is an interference pattern of many motor unit action potentials^7^. Often, the surface EMG signal amplitude underestimates the magnitude output from the motor neurons due to “amplitude cancellation”; however, this discrepancy is not consistent across motor units or contraction conditions^6^. Fine wire EMG (fwEMG) can be used to measure motor unit action potentials more precisely. This invasive method uses a needle to insert a fine wire electrode into the muscle to measure the activity of individual motor units. Fine wire EMG minimizes some of the shortcomings of surface EMG by reducing cross talk and tissue filtering effects; although, it poses alternative limitations such as the invasiveness and selectivity of one or just a few motor units out of hundreds. In the last decade non-invasive methods have been developed to extract individual motor unit action potential activity from high density surface EMG (HDsEMG)^5,8,9^. These approaches often use an array of many sensors over a single muscle in combination with algorithmic approaches to extract the motor unit action potential information from the surface EMG. Thus, it may solve some of the limitations of traditional surface EMG and fine wire EMG approaches. Since the motor unit action potential activity is a 1 to 1 representation of the neural drive to the muscle, this information should provide a better approximation of motor intent than traditional surface EMG approaches^10^. Some studies have shown that this approach was superior for motor intent prediction than traditional methods^11–13^.

It is critical to validate these non-invasive motor unit approximation techniques. One approach is simultaneous measurement of electrical activity by both surface and intramuscular electrodes. However, very few datasets containing both exist due to the difficulty of collecting such data. Furthermore, any datasets that do contain both modalities often do not contain trials from dynamic movements. In typical laboratory EMG studies, isometric contractions are performed wherein the muscle length and joint angles are held constant while the muscle produces force. This mitigates some of the movement artifact and noise but may not represent typical muscle behavior during everyday movement. Thus, the interpretation of neural drive during these experiments may have limited application.

The purpose of this dataset^14^ is to provide a high spatiotemporal resolution representation of the motor control system in dynamic, but controlled, conditions. Data collected simultaneously from within the muscle and from the surface during dynamic muscle contractions can be used to validate methods for extracting neural signals from surface EMG, develop models for predicting joint torque output, or develop classifiers for human movement intent.

**Table 1:** Contraction file naming convention with descriptions.

CONTRACTION_LEVEL	Description
ISOK_30, ISOK_90, ISOK_300	Isokinetic contractions at 30, 90, and 300 deg/sec, respectively. There is no trial number as only 1 trial was collected of each.
ISOT_10, ISOT_25, ISOT_50	Isotonic contractions at 10, 25, and 50% MVC, respectively.
RAH_10, RAH_25, RAH_50	Ramp and Hold (isometric) contractions at 10, 25, and 50% MVC, respectively.
SIN_10, SIN_25, SIN_50	Sine wave (isometric) contractions at 10, 25, and 50% MVC, respectively. There is no trial number as only 1 trial was collected of each.
DFMVIC	Dorsiflexion maximal voluntary contraction. There is only 1 file for each subject which contains 3 contractions.

**Table 2:** Data channel information for fine wire EMG files.

Column	Signal	Units
1	Time	s
2	Torque	Nm or %MVIC
3	Angular Velocity	°/sec
4	Position	° or %ROM
5	Memory	
6	fwEMG 1	mV
7	fwEMG 2	mV
8	fwEMG 3	mV
9	fwEMG 4	mV
10	Synch	mV

**Table 3:** Data channel information for HDsEMG files.

Column	Signal	Units
1	Time	ms
2–127	HDsEMG grid	µV
128	Soleus EMG Proximal	µV
129	Soleus EMG Distal	µV
130	Reference (Medial Epicondyle)	µV
131	Torque	µV
132	Trigger (Synch)	µV
133	Angular Position	µV
134	Angular Velocity	µV

## Methods

### Subjects

Seven subjects between the ages of 18 and 45 with no known metabolic or neurological disorders and no history of surgery, current injury or chronic pain to the dominant foot or leg participated in this study. All procedures were approved by the U.S. Army Research Laboratory Institutional Review Board and all subjects provided their informed consent prior to participation.

### Experimental procedure

The experimental design of this study was such that intramuscular and surface electromyography of the tibialis anterior (TA), as well as ankle joint dynamics could be collected simultaneously while the subject performed different types of muscular contraction. The order of operations for this study was as follows:

1. Subject set up in Biodex isokinetic dynamometer
2. Contraction familiarization and practice
3. High density surface EMG instrumentation
4. Maximal voluntary isometric contractions
5. Intramuscular EMG instrumentation
6. Submaximal contractions
  - Isometric Ramp and Hold
  - Isometric Sinusoidal
  - Isotonic Ramp and Hold
  - Isokinetic
7. Intramuscular EMG removal
8. Maximal isokinetic contractions
9. High density surface EMG removal

### Experimental Set-up

**Fig. 1:**
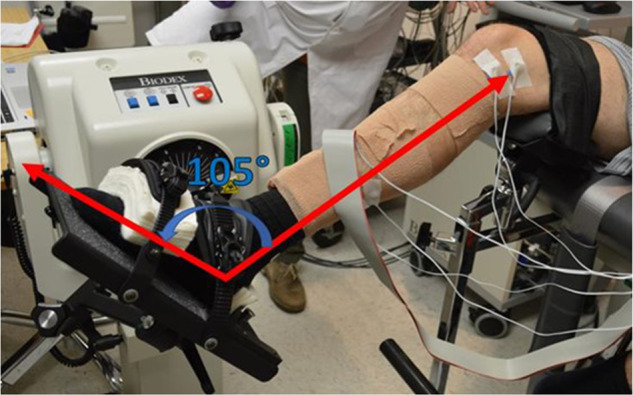
Biodex set up for ankle contraction. Dynamometer motor axis of rotation was aligned with the subject’s lateral malleolus. Red lines indicate alignment between subject’s medial malleolus and medial epicondyle and fifth metatarsal.

For testing, each subject was seated in the Biodex isokinetic dynamometer for ankle dorsiflexion of their dominant leg (Fig. 1). A handheld goniometer was used to set and check lower body joint angles while subject was seated in the dynamometer. Hip flexion was set at 85° using the midaxillary line and the lateral condyle of the femur as landmarks. The knee was supported and allowed to flex at an angle 40° below full extension using the greater trochanter and lateral malleolus as landmarks. This set up was used to not restrict ankle dorsiflexion motion due to tension from the plantarflexor complex (gastrocnemius and soleus) caused by an extended knee. The subject’s foot was strapped to the footplate of the Biodex such that the lateral malleolus was in line with the axis of rotation of the Biodex. The joint angle for the ankle was allowed to vary depending on the contraction type being performed. Restraints were placed over the subject’s shoulders, across the pelvis, and across the contralateral thigh to limit movement compensation. An adjustable table with an LCD monitor was set in front of the Biodex and the height was adjusted such that the monitor was at eye level with the subject. This monitor was used to display real-time feedback in the form of torque or joint position (described in *Contraction Paradigm*).

After the subject was set up according to the specifications above, they were familiarized with the tasks they were to perform and practiced all contractions multiple times until they were comfortable performing them. This was identical to the contraction procedures described in the *Contraction Paradigm* section but done without any instrumentation.

### Instrumentation and acquisition

#### Joint torque, angle, and velocity

Various aspects of joint dynamics of the ankle were collected using the Biodex Isokinetic Dynamometer (Biodex System 4 Pro, Biodex, Shirley, New York, USA). The dynamometer measures the amount of torque that is exerted on a motor through a level arm connected to the head of the dynamometer (Fig. 1). The Research Toolkit (RTK) software on the Biodex system allows access to raw analog output of torque, angular position, and angular velocity (±5 V, 1000 Hz). The scaling factors for torque, position, and velocity per 1 V output were 34.78 Nm, 34.68°, and 25.575 deg/sec, respectively.

#### High density surface electromyography

**Fig. 2:**
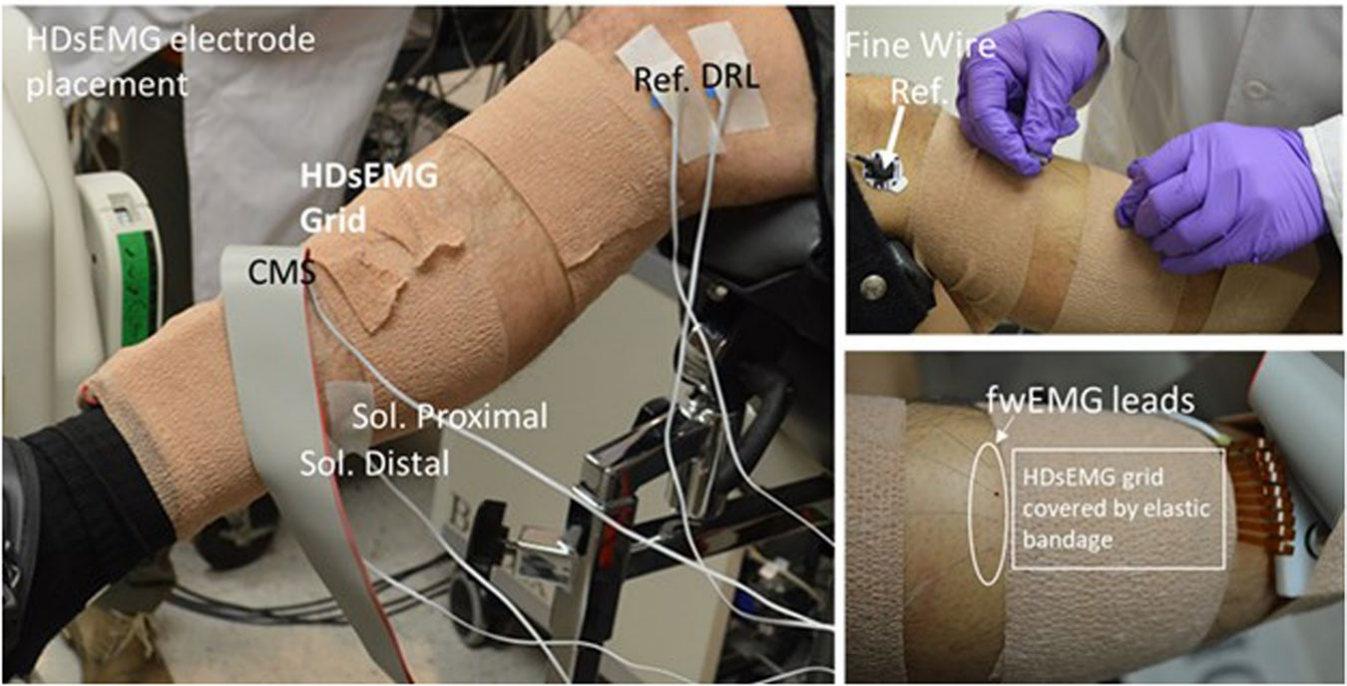
Locations of sensors. Note in the top right pane that the fwEMG leads were inserted at a 45 deg angle such that the ends of the fine wire electrodes would be embedded in the muscle fibers just below the HDsEMG grid.

High density surface electromyography (HDsEMG) was collected via a thin film substrate with 126 channels in a 9 × 14 grid with a center-center distance of 4 mm (Biosemi, Amsterdam, Netherlands). Each electrode was round (1.95 mm in diameter) and was constructed of a copper body coated with pure sliver (99.99% Ag). We prepared the skin for surface EMG electrodes by dry shaving with a disposable razor to remove hair and dead skin. Next, we scrubbed the skin with an alcohol swab to further clean and debride. We centered the HDsEMG grid over the belly of the tibialis anterior muscle (seniam.org), leaving enough room proximal to the grid for fine wire electrode insertion. The 126 electrodes formed a rectangular grid. The long side of the grid was placed in parallel with the muscle fibers. We coated each electrode with a small amount of electrolyte paste and attached the grid to the skin using a double-sided sticker with perforations for each electrode. We covered the HDsEMG grid with elastic bandage to ensure good contact with the skin. We also recorded muscular activity of the soleus muscle on the same leg by placing two monopolar EMG electrodes (4 mm Ag/AgCl active electrodes, Biosemi, Amsterdam, Netherlands) in series along the medial aspect of the soleus muscle. Additionally, a single Ag/AgCl active electrode was placed on the medial epicondyle for use as a reference electrode if desired (Fig. 2). Note that the Biosemi ActiveTwo system collects the data unreferenced and thus the user can select the desired reference in post processing. The Biosemi ActiveTwo system uses common mode sense (CMS) and driven right leg (DRL) sensors instead of common ‘ground’ electrodes. The CMS was placed distal to the thin film HDsEMG grid near the tibial plateau. The DRL was applied to the medial epicondyle of the same leg.

#### Intramuscular electromyography

Intramuscular neural activity of the tibialis anterior was collected via four (4) fine wire electrodes (Chalgren Enterprise, Gilroy, California, USA). Each fine wire electrode was composed of two insulated stainless-steel wires 0.51 mm in thickness. No insulation was removed from the tip of each wire such that only the cross-section area of the end of the wire was exposed. This exposure area was chosen after initial piloting of fine wires with 5 mm exposure areas revealed exceedingly noisy signal during movement, making motor unit action potential waveforms difficult to discern. Each fine wire was inserted approximately parallel to the muscle fibers at a 45° angle from the surface of the skin via a 27-gauge hypodermic needle. The wires were inserted just proximal to the HDsEMG grid such that the tip of the wires sat in the muscle belly underneath the HDsEMG grid. A pre-gelled Ag/AgCl adhesive ground electrode was placed on the participant’s lateral condyle (Fig. 2). Exposed ends of the wires were connected to the bipolar bioamplifier (B&L Engineering, Santa Ana, CA) where data was then transmitted to the data acquisition system (detailed in *Data Acquisition and Synchronization)*. All fwEMG signals were pre-amplified with a 330× gain. The placement of all electrodes were confirmed by asking the subject to perform light dorsiflexion contractions and visually inspecting electromyography signal.

#### Data acquisition and synchronization

Fine wire EMG and Biodex data were collected simultaneously with the Micro1401-3 data acquisition system (Cambridge Electronic Design Limited, Cambridge, UK) using the Spike2 version 9.0 (Cambridge Electronic Design Limited, Cambridge, UK) data acquisition software. Fine wire data was sampled at 10 KHz while Biodex data was sampled at 2048 Hz. HDsEMG data was sampled at 2048 Hz using the BioSemi ActiveTwo biopotential amplifier and recorded using ActiView software provided by the manufacturer (Biosemi, Amsterdam, Netherlands). The Biosemi Analog Input Box (AIB) was daisy chained with the Biosemi ActTwo amplifier to allow for simultaneous recording of HDsEMG and Biodex data channels (split for recording by each system). Prior to entering the Biosemi AIB, each of the Biodex analog outputs (torque, position, and velocity) included a voltage drop (1/5) to ±1 V to accommodate the Biosemi ADC range. For data alignment between the Micro 1401-3 and Biosemi data acquisition systems, a 1 V double square wave synch pulse was output from the Micro1401-3 system to the Biosemi AIB. The 1 V square waves were sent prior to each trial recording to enable post hoc time synchronization.

### Contraction paradigm

#### Maximal voluntary isometric contraction (MVIC)

For MVIC contractions, the subject was not given any visual feedback. The footplate was locked in a position of 15° plantarflexion. The subject was asked to “pull their toes toward their shin” against the stationary footplate for 3–5 seconds. After practicing at submaximal forces, three to five similar maximal contractions were obtained sequentially in order to generate a reliable estimate of maximal torque production. Strong positive verbal encouragement was given during the contraction to elicit reliable measurement. These contractions are separated by a minimum of 60 seconds.

#### Torque normalization

The maximum torque produced during the MVIC’s was used to scale all subsequent contractions to a percentage intensity, or %MVIC, such that cross subject and condition comparisons can be made. Briefly, after the subject was set up for ankle dorsiflexion in the Biodex, the subject was asked to remain still and relaxed as a baseline was collected. The average voltage produced during a 1 second interval was calculated and used at the gravity offset labeled “Offset”. Once the MVIC’s were complete, the max value achieved was designated as a 100% effort of the contraction. The difference between this maximum and offset values is considered the “Scale” representing 0–100% of the MVIC.

#### Torque conversion between biodex and biosemi

Metadata relating to each subject’s individual calculation of %MVIC is provided on Open Science Framework as “Instructions and Values for Biosemi Torque (uV) to Biodex Torque (%MVIC) Conversion.” Provided below is the formula for deriving %MVIC from the Biosemi system torque data, accounting for unit difference and voltage drop (Eq. 1).

1 $$Biosemi\;{\rm{ \% }}MVIC=\left(\left(\frac{{\rm{\mu }}V}{1{e}^{6}}\right)* 5* Scale\right)+Offset$$

*Scale* and *Offset* refer to the percentage of MVIC per 1 V produced (at the Micro1401-3) and the negative effect of gravity, respectively.

#### Isometric ramp and hold contractions

**Fig. 3:**
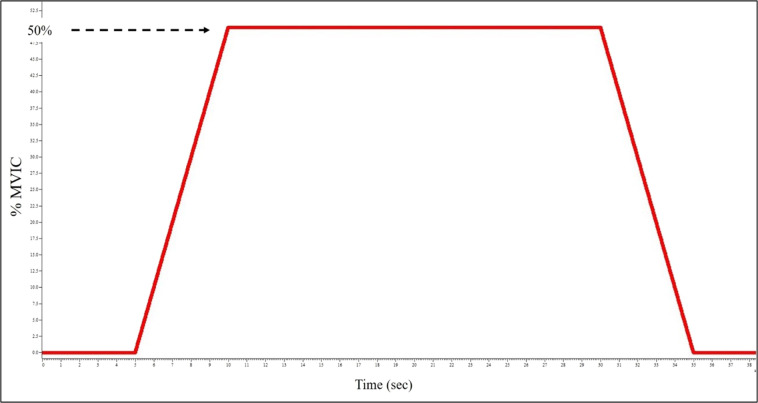
Template used for visual feedback by subjects to match torque ramp and hold during isometric contractions.

Subjects were asked to perform isometric ramp and hold contraction at three different intensity levels, 10, 25, and 50% MVIC. Three to five contractions were performed at each intensity. Similar to the MVIC task, the footplate was locked in 15° plantarflexion. Real-time torque feedback was displayed to the subject over a trapezoidal template, and they were instructed to trace the line by slowly pulling up with their toes (Fig. 3). The slope was standardized for all subjects and intensity levels such that they were required to produce +5% per second to ramp up and −5% per second on the ramp down. The constant torque section was held for 20 seconds. A rest of 60 seconds was given between contractions.

#### Isometric sinusoidal contractions

**Fig. 4:**
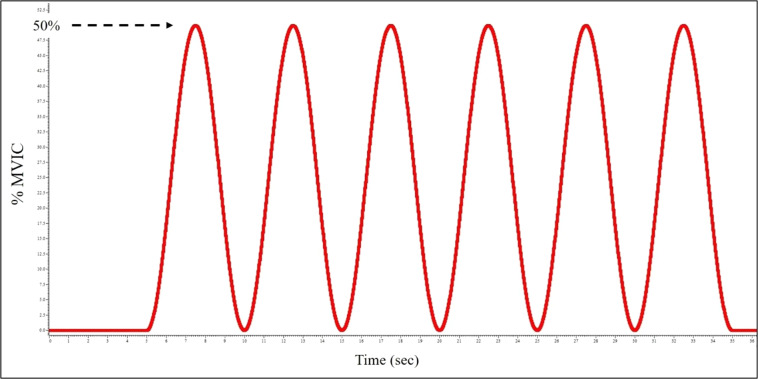
Template used for visual feedback by subjects to match torque sinusoid during isometric contractions.

The set up and instructions for the isometric sinusoidal contractions were similar to that of the ramp and hold contractions. However, the subject was instructed to trace a sinusoidal wave template between 0 and the prescribed intensity level (Fig. 4). The peak torque produced during the wave was set for 10, 25, 50% MVIC, similar to the ramp and hold contractions. Pilot testing revealed that a waveform with frequency of 0.2 Hz for a duration of 30 second (6 cycles) was feasible for smooth torque traces at all three intensity levels. A single trial was recorded for each intensity level. Subjects were given 60 seconds of rest between contractions.

#### Isotonic contractions

**Fig. 5:**
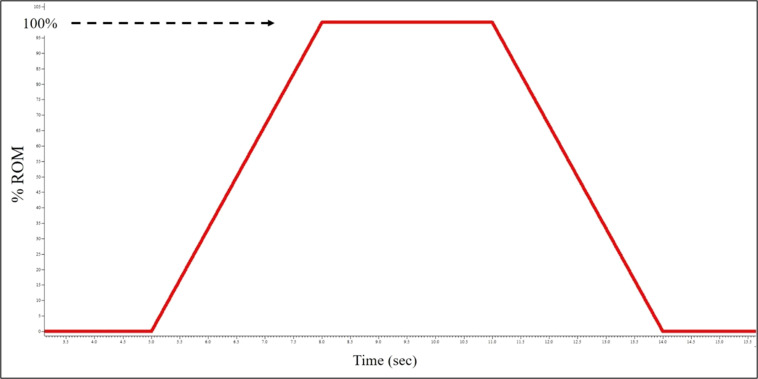
Template used for visual feedback by subjects to match position ramp and hold during isotonic contractions.

The isotonic contractions consisted of the subject rotating the footplate with their ankle through a prescribed range of motion while the dynamometer induced a prescribed constant resistance. Pilot testing revealed that a range of motion of 10° dorsiflexion to 20° plantarflexion was feasible to produce smooth contractions. Pilot testing also revealed real-time visual feedback using instantaneous position produced visually smoother traces than torque in isotonic contractions. The template each subject was asked to trace was similar to the template used for the ramp and hold contractions; however, in these testing iterations the Y-axis displayed position. The isotonic contractions consisted of a ramp up, hold, and ramp down phase (Fig. 5). The intent was to produce a constant torque throughout all phases.

**Fig. 6:**
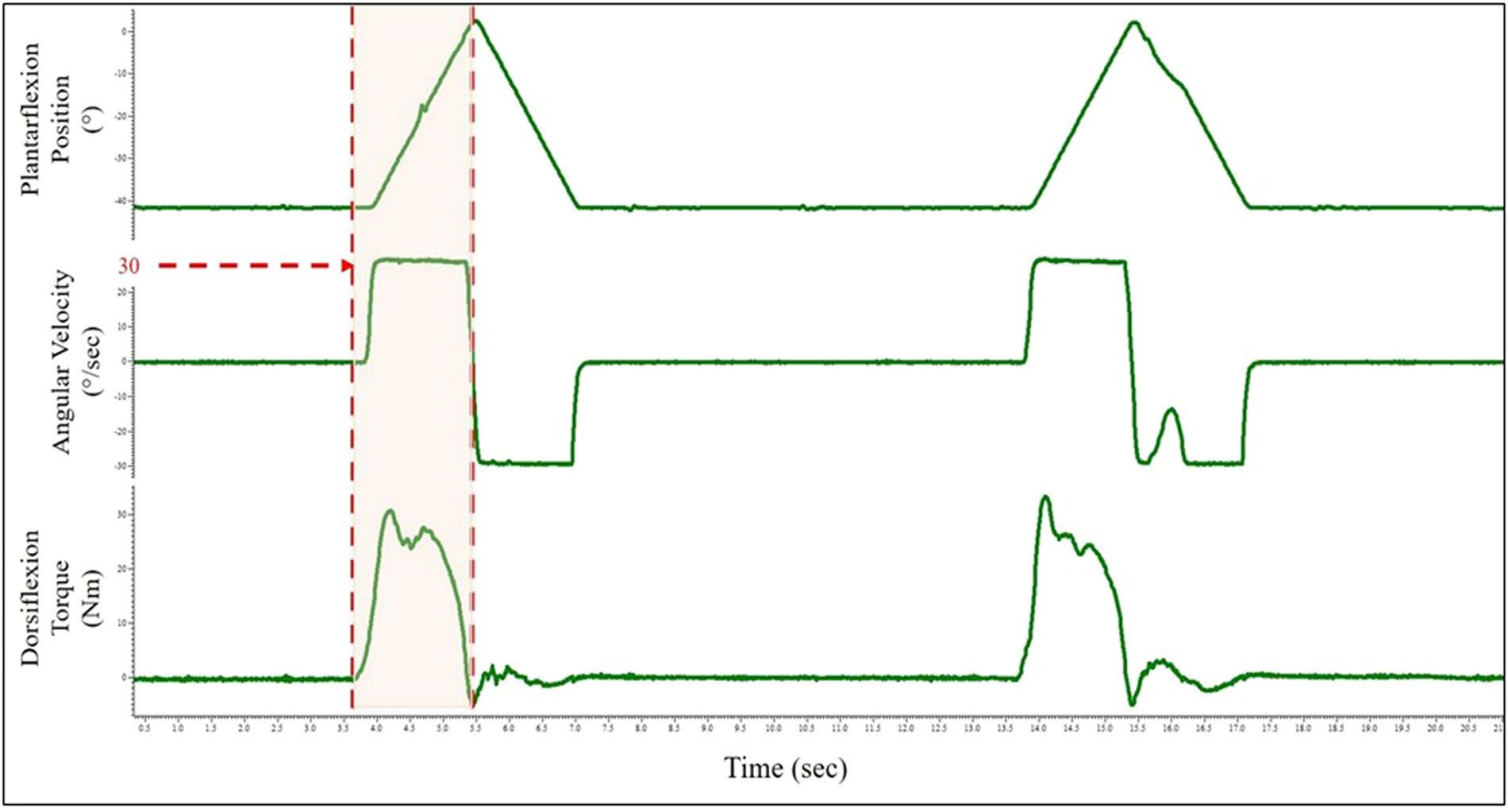
Sample output of 2 out of the 5 isokinetic contractions at 30°/ sec. Ankle Position (top), Angular Velocity (middle), and Torque (bottom) are shown with the shaded region indicating the isokinetic dorsiflexion phase of the contraction. Note: Angular Velocity is seen to plateau at 30°/ sec where the dynamometer added resistance, with the subsequent increase in torque.

**Fig. 7:**
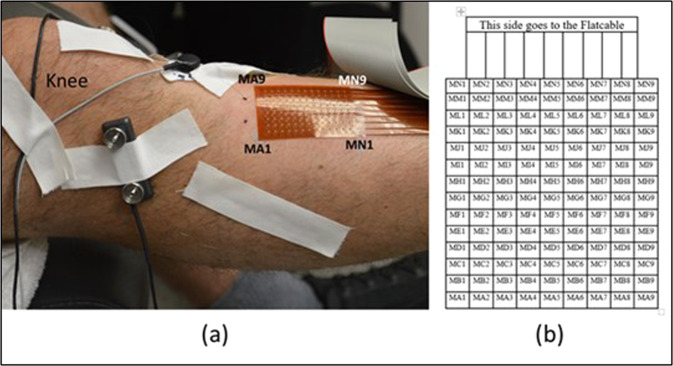
HDsEMG grid placement orientation (**a**) and channel layout (**b**). Note that in (**b**) the electrode contacts are facing upward.

The ramp up phase consisted of the subject beginning at rest at 20° plantarflexion. They were instructed to pull their toes toward their shins in a smooth and controlled manner at a rate of 10° per second while the dynamometer resisted movement. This was the concentric phase of the contraction as the muscle length decreased during this phase over a constant torque output. Once at the end of the range of motion, the subject would hold the steady torque at that position for three seconds. This was the hold phase of the contraction.

The ramp down phase consisted of the subject beginning at 10° dorsiflexion and letting the torque produced by the dynamometer rotate their angle back toward plantarflexion in a slow and controlled motion. Again, the subject was asked to maintain a speed of −10° per second (~3 seconds) for this phase. This was considered the eccentric phase of the contraction as the muscle length were increasing against a constant resisting force (the dynamometer). Three to five trials were conducted at prescribed torque levels of 25% and 50% MVIC.

#### Isokinetic contraction

Once all fine wire instrumentation was removed from the subject, isokinetic contractions were performed. These contractions were performed with a maximal effort through an unconstrained range of motion, mimicking very dynamic movements experienced in sport and daily living. For isokinetic contractions, torque imparted on the joint was due in part to velocity limitations set by the dynamometer. For contractions with equal contraction intensity (e.g., maximal effort contractions), a maximum angular velocity setting that is low (e.g., 30° per second) will provide more resistance and induce higher torque levels at the joint compared to higher angular velocities (e.g., 300° per second). In cases where the maximum angular velocity is very high and the range of motion is narrow, it can be difficult for subjects to reach that speed. As such, the 300° per second condition is practically a maximum attainable velocity.

First, a prescribed maximum angular velocity was set for the dynamometer. The subject began each trial in a relaxed plantarflexed position (~25°) and were instructed to pull their toes to their shin using maximal effort as the footplate rotated. Once they reached the end of their range of motion (~15°) in a dorsiflexed position, they were instructed to relax their foot back down to starting position. This procedure was repeated for 5 contractions within a single trial, with 10–15 seconds recovery between contractions. To vary the torque levels experienced, one trial was performed at each 300° (or maximal), 90°, and 30° per second velocities. No visual feedback was given during the isokinetic trials.

## Data Records

All data files are publicly available through Open Science Framework and are de-identified (10.17605/OSF.IO/9S3U6)^14^. All subjects gave their informed consent to share their de-identified data. The data are archived in multiple file sets containing metadata, HDsEMG/Biodex data and fwEMG/Biodex data. On the OSF data repository, fine wire EMG data is located in the directory ‘fwEMG’ and high-density surface EMG data is located in the ‘hdEMG’ directory. Within each of those directories, there is a sub-directory for each subject’s data. For example directory ‘S01’ is subject 1 data only. Within each subject’s directory, each condition and trial are a separate file. For example, trial 1 ramp and hold 25%MVC condition for subject 1 is ‘S01_RAH_25_1.csv’. The HDsEMG and fwEMG files have the following naming convention:

SUBJECT#_CONTRACTION_LEVEL_TRIAL#.txt

Further details about file naming convention can be found in Table 1. Tables 2 and 3 provide detailed description of the content of each file, including channel types and units.

### Fine wire EMG

For the DFMVIC and ISOK fwEMG files, torque is reported in Newton-Meters (Nm), whereas for all other file types it is scaled to units of %MVIC. For ISOT fwEMG files, position is recorded as %ROM, whereas all other file types it is scaled to absolute degrees. Angular velocity is recorded as degrees per sec (deg/sec) in all files. The memory channel contains the torque or position template that is given as visual feedback to the subjects to track and match their torque or position to.

Because the MVIC and isokinetic contractions were maximum effort, fine wire EMG was not included in the recording. These files only contain the Torque, Angular Velocity, Position, and Synch channels. NOTE: Subject 1 contains different channel number information due to testing of bipolar surface electrodes. However, channel *names* are still consistent.

## Technical Validation

The quality of the fwEMG signals is greatly affected by electrical noise and movement artifact which can distort the individual motor unit action potentials within the signal. Proper grounding and skin preparation measures were taken. Light isometric contractions (~10% MVC) were performed to visually assess the fwEMG signal after insertion of all the wires. If motor unit action potentials were not seen, then either the wire was shifted manually by the operator, or the muscle was lightly massaged to try to get the exposed end of the wire on a firing muscle fiber.

**Fig. 8:**
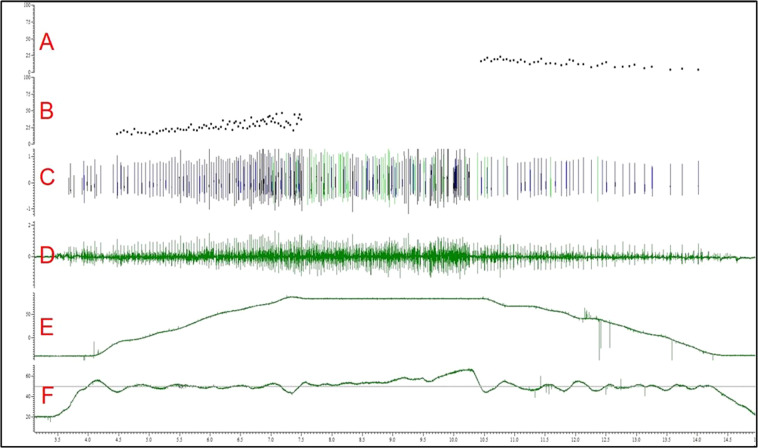
Example motor unit analysis from a 50% MVIC isotonic contraction. Panel (**A**) inter-spike intervals for eccentric contraction phase, (**B**) inter-spike interval for concentric contraction phase, (**C**) waveform template matching spiking, (**D**) filtered fine wire EMG signal, (**E**) ankle position, (**F**) dorsiflexion torque.

For theoretical validation of fwEMG signal quality, the data from a representative isotonic trial was decomposed into motor unit action potentials with a waveform template matching algorithm with Spike2. As displayed in Fig. 8, the firing rates during the concentric phase (Panel B) are greater than those during the eccentric phase (Panel A). Eccentric contractions elicit lower firing rates at the same torque level, due to the elastic and inelastic properties of the muscle-tendon unit^15,16^. We see this pattern in a sample of our isotonic data, demonstrating that the fwEMG during dynamic contractions is of sufficient quality for standard analysis approaches.

The quality of the HDsEMG signal was first assessed when placing the electrodes by measuring the voltage offsets from BioSemi’s Actiview software and ensuring the offsets were below the manufacturer recommended levels. Further, the data were visually inspected in real time to ensure appropriate signal response during a muscle contraction and that there weren’t any unusual artifacts or excessive 60 Hz line noise. If poor signal quality was detected, the electrode grid was removed and reapplied before data acquisition. During data acquisition, the data were continually monitored for excessive artifacts. Some artifact was expected, especially during the higher rate isokinetic trials.

**Fig. 9:**
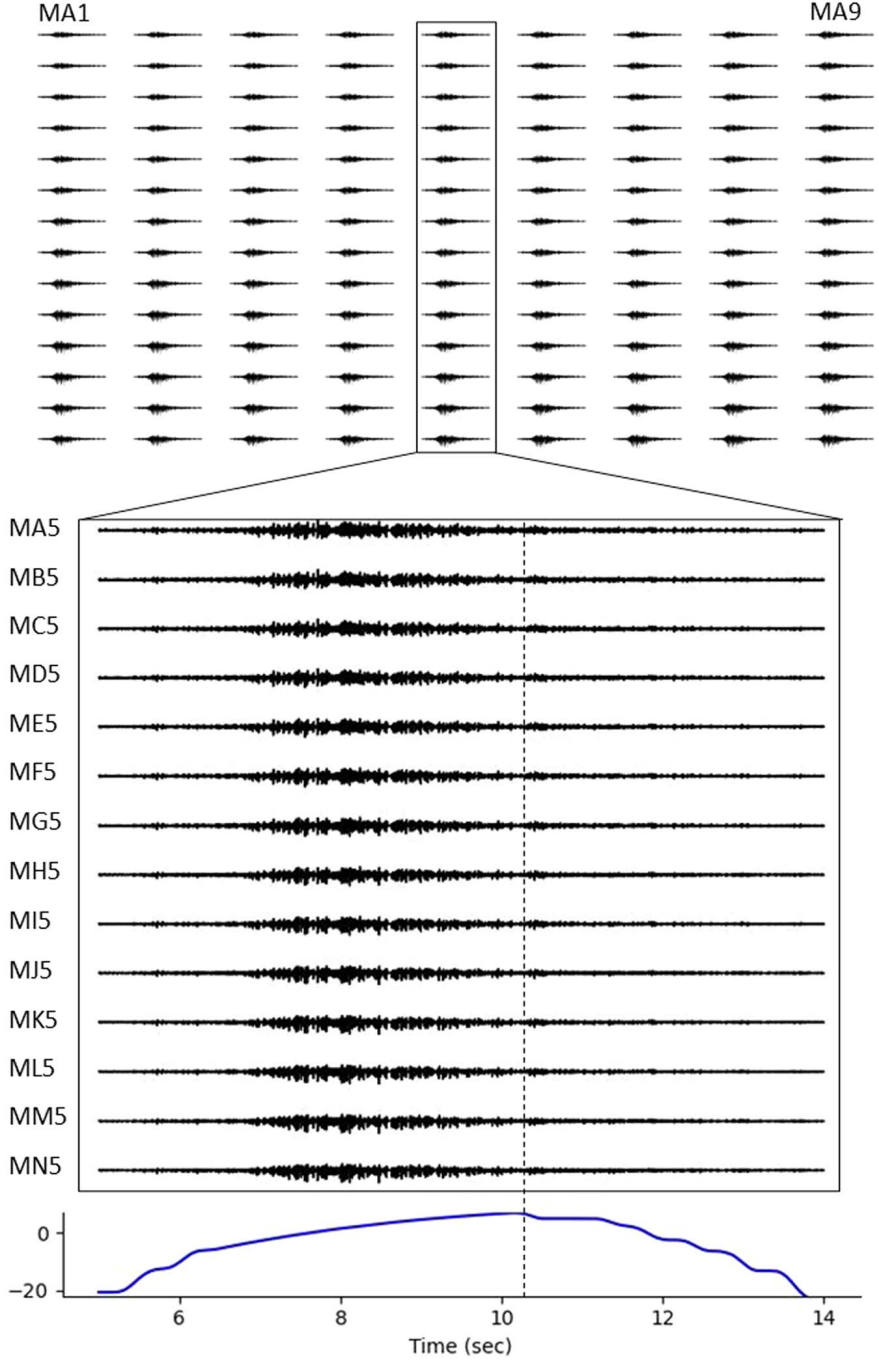
Minimally processed HDsEMG data from a representative isotonic trial. Data were band pass filtered 20–400 Hz for visualization purposes. HDsEMG traces are plotted for the full grid of 126 channels. For more detail, we zoomed in for a single column of 14 channels plotted along with sagittal plane, ankle joint angle. The vertical dashed line roughly denotes the transition to eccentric contraction.

Minimally processed HDsEMG data for one representative isotonic trial is presented in Fig. 9. For visualization only, the data were filtered between 20–400 Hz, primarily to remove DC offset and any slow drifts in the data. Data quality is demonstrated by the signal consistency across all 126 channels showing a similar increase and decrease in activity as the subject moves through the concentric, isometric, and eccentric phases of the isotonic trial. In Fig. 9, we further zoom in on one column of data plotted along with the ankle joint angle in degrees. In this part of Fig. 9, we demarcate the transition to eccentric contraction with a vertical dashed line to highlight the expected, relative decrease in EMG activity.

Data quality is further demonstrated by the results presented in two publications based on the analysis of data summarized in the current publication^17,18^. In Leahy^18^, we utilized existing algorithms^8^ to decompose the HDsEMG into motor unit spike trains and then used rate coding and kernel smoothing approaches to estimate torque output during the concentric portion of the isotonic trials. We used pulse to noise ratio (PNR) to identify sources that were representative of motor unit spike trains^19^. We found that we were able to decompose motor unit spike train sources from the HDsEMG during dynamic contractions using the aforementioned blind source separation approaches and we were able to use the composite spike trains to estimate ankle dorsiflexion torque. Addtionally, in^17^, the decomposition of the HDsEMG into motor unit spike trains for a broad range of conditions from this dataset are presented. For example, HDsEMG from the isometric sinusoidal contractions was decomposed into motor unit spike trains. The spike trains during the sinusoidal contractions were found to increase and decrease activity in a pattern that corresponds with the measured output torque as depicted in Figs. 4–8 from^17^.

HDsEMG data from the isokinetic trial conditions have not yet been analyzed or published. However, the data was collected during the same sessions as the previously analyzed and presented data.

## Usage Notes

Both fwEMG and HDsEMG are prone to movement artifact during muscle contraction, even during isometric contractions. Furthermore, the fwEMG wires within the muscle belly could shift and start recording a different muscle fiber. Additionally, the sliding of the muscle underneath the skin on which the surface electrodes are placed may functionally change the recording area for HDsEMG. Simultaneously recording these along with torque and position gets us close to matching electrical changes to morphological changes. However, without imaging like ultrasound, we cannot quantitatively validate this.

The TA is not the only lower leg muscle activated during dorsiflexion. While palpation of the muscle belly and manual checking were done when placing the HDsEMG electrode grid, cross-talk may occur. Cross-talk is when activation of neighboring muscles is picked up by the recording electrodes. Activation of the Extensor Digitorum Longus or the Extensor Hallucis Longus may be present in the HDsEMG signal.

## Data Availability

No custom code was used to administer the experimental paradigm or collect data. The data stored in the repository are raw data and has not been processed with custom code. Some sample custom scripts to load and visualize data are saved with the data in the Open Science Framework repository (10.17605/OSF.IO/9S3U6).
